# Crystal structure of bis­{2-[5-(3,4,5-tri­meth­oxyphenyl)-4*H*-1,2,4-triazol-3-yl]pyridine}palladium(II) bis­(tri­fluoro­acetate) tri­fluoro­acetic acid disolvate

**DOI:** 10.1107/S205698902400392X

**Published:** 2024-05-03

**Authors:** Borys V. Zakharchenko, Dmytro M. Khomenko, Roman O. Doroshchuk, Alexandra Bargan, Olga Yu. Vassilyeva, Rostyslav D. Lampeka

**Affiliations:** a Department of Chemistry, Taras Shevchenko National University of Kyiv, Volodymyrska str. 64/13, 01601 Kyiv, Ukraine; b Enamine Ltd. (www.enamine.net), Winston Churchill str. 78, 02094 Kyiv, Ukraine; c "PetruPoni" Institute of Macromolecular Chemistry, Aleea Gr., Ghica Voda 41A, 700487 Iasi, Romania; Universidad de Los Andes, Venezuela

**Keywords:** crystal structure, Pd^II^ complex, 3-(pyridin-2-yl)-1,2,4-triazole, tri­fluoro­acetate anion, hydrogen bonding

## Abstract

The new palladium(II) complex, [Pd(C_16_H_16_N_4_O_3_)_2_](CF_3_COO)_2_·2CF_3_COOH, crystallizes in the triclinic space group *P*  with the asymmetric unit containing half the cation (Pd^II^ site symmetry *C_i_*), one tri­fluoro­actetate anion and one co-crystallized tri­fluoro­acetic acid mol­ecule. Two neutral chelating 2-[5-(3,4,5-tri­meth­oxy­phen­yl)-4*H*-1,2,4-triazol-3-yl]pyridine ligands coordinate to the Pd^II^ ion through the triazole-N and pyridine-N atoms in a distorted *trans*-PdN_4_ square-planar configuration [Pd—N 1.991 (2), 2.037 (2) Å; *cis* N—Pd—N 79.65 (8), 100.35 (8)°]. The complex cation is quite planar, except for the methoxo groups (δ = 0.117 Å for one of the C atoms). The planar configuration is supported by two intra­molecular C—H⋯N hydrogen bonds. In the crystal, the π–π-stacked cations are arranged in sheets parallel to the *ab* plane that are flanked on both sides by the tri­fluoro­acetic acid–tri­fluoro­acetate anion pairs. Apart from classical N/O—H⋯O hydrogen-bonding inter­actions, weak C—H⋯F/N/O contacts consolidate the three-dimensional architecture. Both tri­fluoro­acetic moieties were found to be disordered over two resolvable positions with a refined occupancy ratio of 0.587 (1):0.413 (17) and 0.530 (6):0.470 (6) for the protonated and deprotonated forms, respectively.

## Abstract 2

In the Pd^II^ complex, two substituted 3-(pyridin-2-yl)-1,2,4-triazole ligands in the neutral form coordinate to the metal atom through the pyridine-N and triazole-N atoms in a *trans*-configuration.

## 1. Chemical context

Triazoles are five-membered heterocyclic compounds containing three nitro­gen atoms and two carbon atoms in the ring. They can exist in different isomeric forms, such as 1,2,3-triazole and 1,2,4-triazole. 1,2,4-Triazole derivatives are of inter­est in various research fields ranging from medicinal chemistry and pharmaceuticals (Aggarwal & Sumran, 2020 ▸; Leenders *et al.*, 2021 ▸) to materials science (Farooq, 2020 ▸). Versatile coordination behaviour due to the presence of neutral, anionic or cationic nitro­gen donors (N-coordination) as well as carbanionic donors (C-coordination) makes 1,2,4-triazoles appealing ligands for the construction of metal complexes with useful functionalities (Song *et al.*, 2019 ▸; Feltham *et al.*, 2017 ▸; Kumar *et al.*, 2015 ▸; Wen *et al.*, 2017 ▸). Substitution reactions at the azole ring create a virtually unlimited range of chemical and structural variations to tune the desired characteristics of the resulting complexes.

In our ongoing project exploring the rich potential of 1,2,4-triazoles in coordination and supra­molecular chemistry, a number of new metal complexes bearing 3-(pyridin-2-yl)-1,2,4-triazole derivatives as ligands were prepared. The Cu^II^, Ru^II^, Pd^II^, Eu^III^, Tb^III^ and Pt^II^ compounds revealed promising magnetic (Petrenko *et al.*, 2021 ▸), catalytic (Zakharchenko *et al.*, 2019 ▸) and luminescent properties (Khomenko *et al.*, 2015 ▸, 2023 ▸), as well as anti­proliferative activity against several human cancer cell lines (Ohorodnik *et al.*, 2022 ▸, 2023 ▸). 
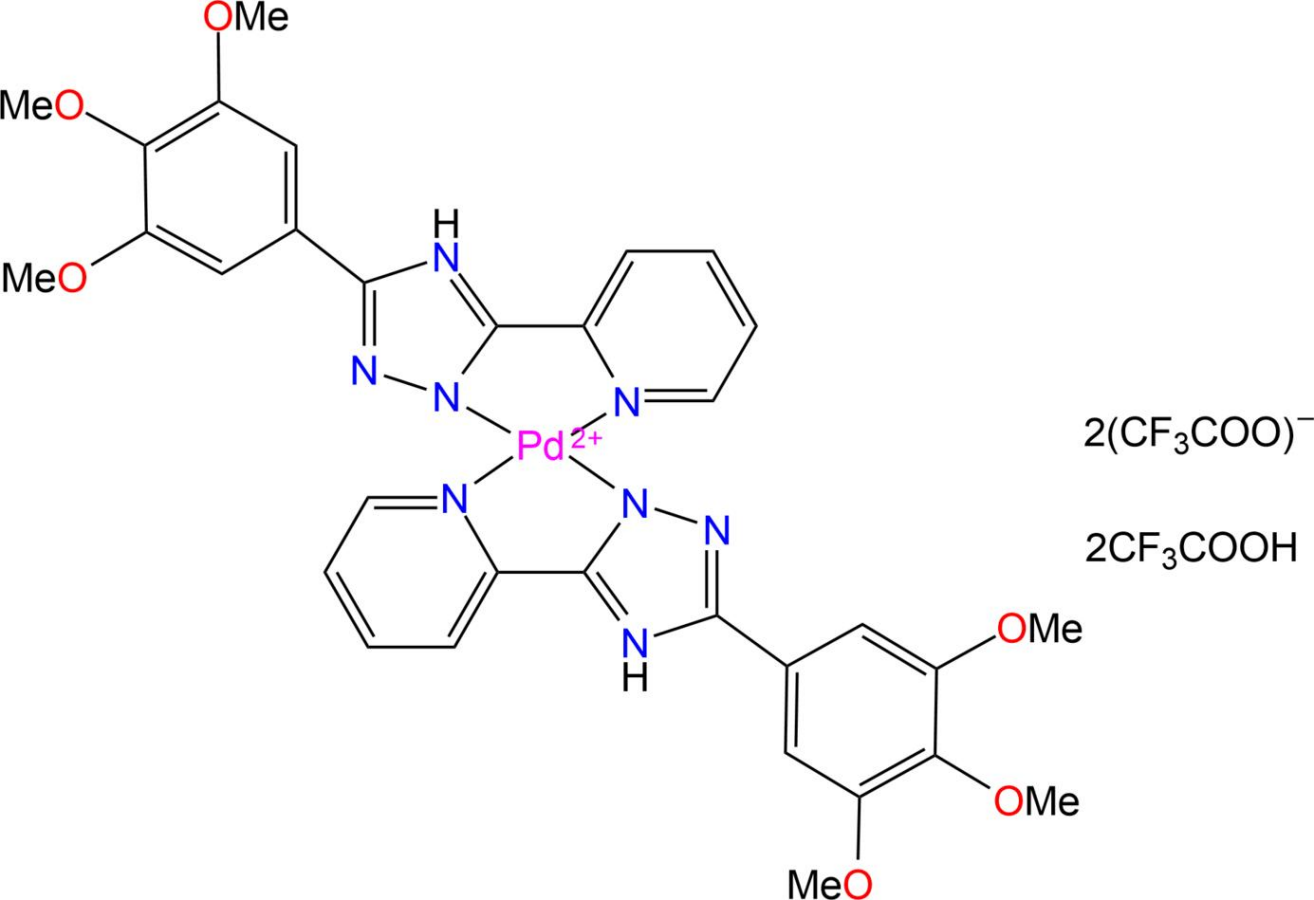


In the present study, the crystal structure of [Pd(H*L*)_2_](CF_3_COO)_2_·2CF_3_COOH, (I), where H*L* is 2-[5-(3,4,5-tri­meth­oxy­phen­yl)-4*H*-1,2,4-triazol-3-yl]pyridine, is reported. The title compound was isolated in an attempt to recrystallize its neutral precursor Pd*L*_2_ from tri­fluoro­acetic acid (TFA). Pd*L*_2_ was prepared and studied with IR, UV–Vis, NMR and photoluminescence spectroscopy, as well as MALDI mass spectrometry in solution and solid state but not structurally characterized (Zakharchenko *et al.*, 2016 ▸).

## 2. Structural commentary

**Figure 1:**
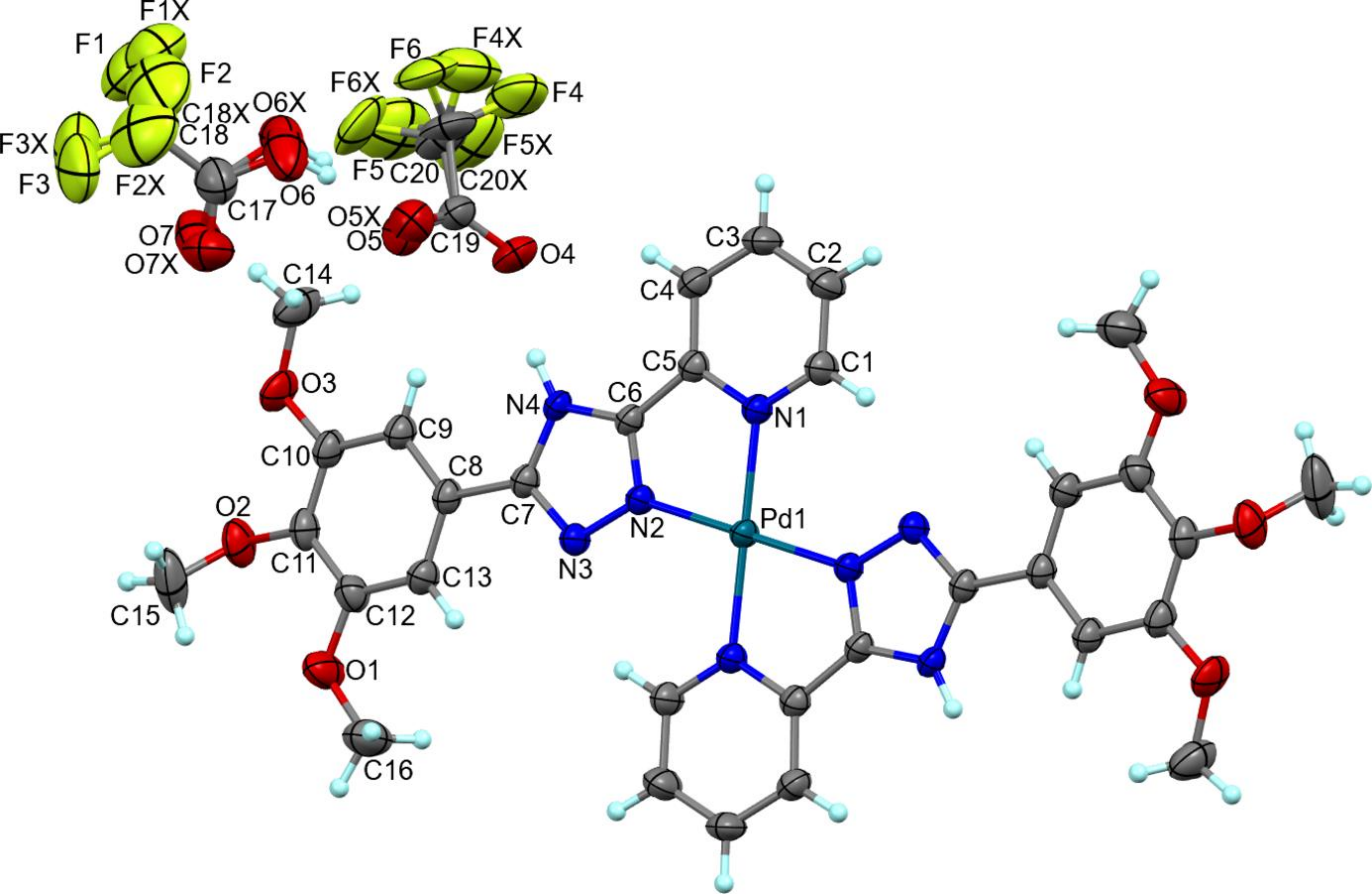
Extended view of the asymmetric unit of (I) with the atom labelling and displacement ellipsoids at the 50% probability level showing the coordination environment of the metal atom.

**Figure 2:**
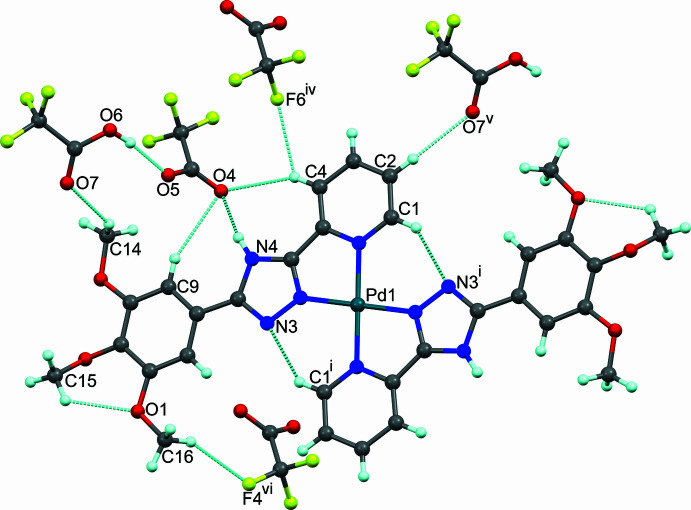
Intra­molecular C1—H1⋯N3^i^ and C15—H15*B*⋯O1 hydrogen-bonding inter­actions forming rings of *S*(6) graph-set motif and inter­molecular hydrogen bonds involving the tri­fluoro­acetic moieties of (I) (blue dashed lines). Minor disorder components have been omitted for clarity. Symmetry codes as given in Table 2 ▸.

The title compound is assembled from discrete [Pd(H*L*)_2_]^2+^ cations (the Pd^II^ atom is located on a special position with *C_i_* site symmetry), CF_3_COO^−^ anions, and CF_3_COOH mol­ecules of crystallization in a 1:2:2 ratio (Fig. 1 ▸). Both neutral H*L* mol­ecules are coordinated to the metal atom as bidentate ligands through the triazole-N2 and pyridine-N1 atoms in a *trans*-configuration. The square-planar N_4_ environment of the Pd^II^ centre is moderately distorted with the two Pd—N distances and two *cis* N—Pd—N angles differing by 0.046 (2) Å and 20.70 (8)°, respectively (Table 1 ▸). The [Pd(H*L*)_2_]^2+^ cation, except for the methoxo groups, is almost planar with the largest deviation from the mean plane being 0.117 Å (C11). Two intra­molecular hydrogen bonds, C1—H1⋯N3^i^ and C15—H15*B*⋯O1, with an *S*(6) graph-set motif are observed (Fig. 2 ▸, Table 2 ▸; symmetry code as given in Table 2 ▸) (Etter, 1990 ▸). The C—H⋯N *S*(6) rings support the planar configuration of the cation.

The C—O bond distances for disordered carb­oxy­lic [1.177 (7)/1.174 (8), 1.273 (7)/1.275 (8) Å] and carbox­ylate units [1.223 (4), 1.227 (11)/1.231 (13) Å] unequivocally confirm the mol­ecular and anionic forms of the TFA and TFA^−^ anion, respectively.

## 3. Supra­molecular features

**Figure 3:**
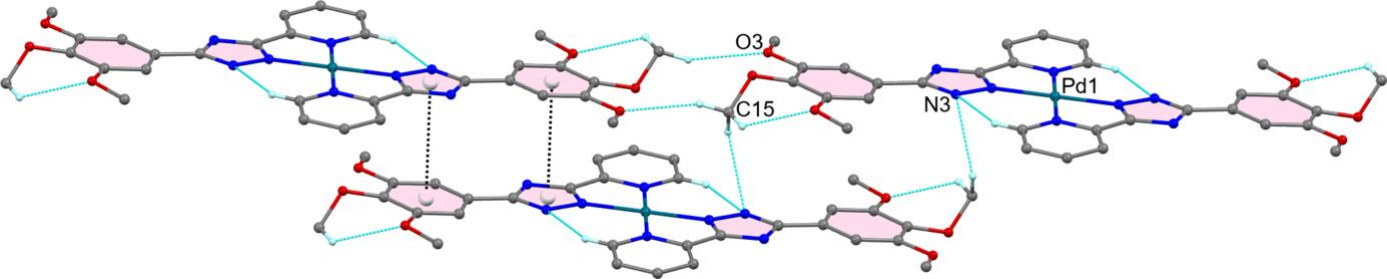
[Pd(H*L*)_2_]^2+^ cations of (I) joined by aromatic stacking between triazole and benzene rings of the centrosymmetrically related ligands (black dashed lines) and inter­molecular C15—H15*A*⋯N3^ii^ and C15—H15*C*⋯O3^iii^ inter­actions forming rings with 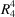 (18) and 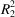 (12) graph-set motifs (blue dashed lines). H atoms not involved in hydrogen bonding have been omitted for clarity. [Symmetry codes: (ii) −*x* + 1, −*y* + 1, −*z* + 1; (iii) −*x* + 1, −*y*, −*z* + 1.]

The crystal structure is built up of an alternate arrangement of distinct cationic and anionic supra­molecular layers oriented in the *ab* plane. In the cationic layer (Fig. 3 ▸), the face-to-face aromatic stacking between triazole and benzene rings of the centrosymmetrically related ligands is significantly offset, as evidenced by a centroid-to-centroid distance of 3.566 (2) Å with the inter­planar distance and tilt angle being 3.263 Å and 76.26°, respectively. The [Pd(H*L*)_2_]^2+^ cations are additionally inter­twined by weak C15—H15*A*⋯N3^ii^ and C15—H15*C*⋯O3^iii^ hydrogen-bonding inter­actions (Table 2 ▸; symmetry codes as given in Table 2 ▸) forming rings of 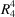 (18) and 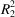 (12) graph-set motifs. The closest Pd⋯Pd separation in the layer exceeds 10 Å.

**Figure 4:**
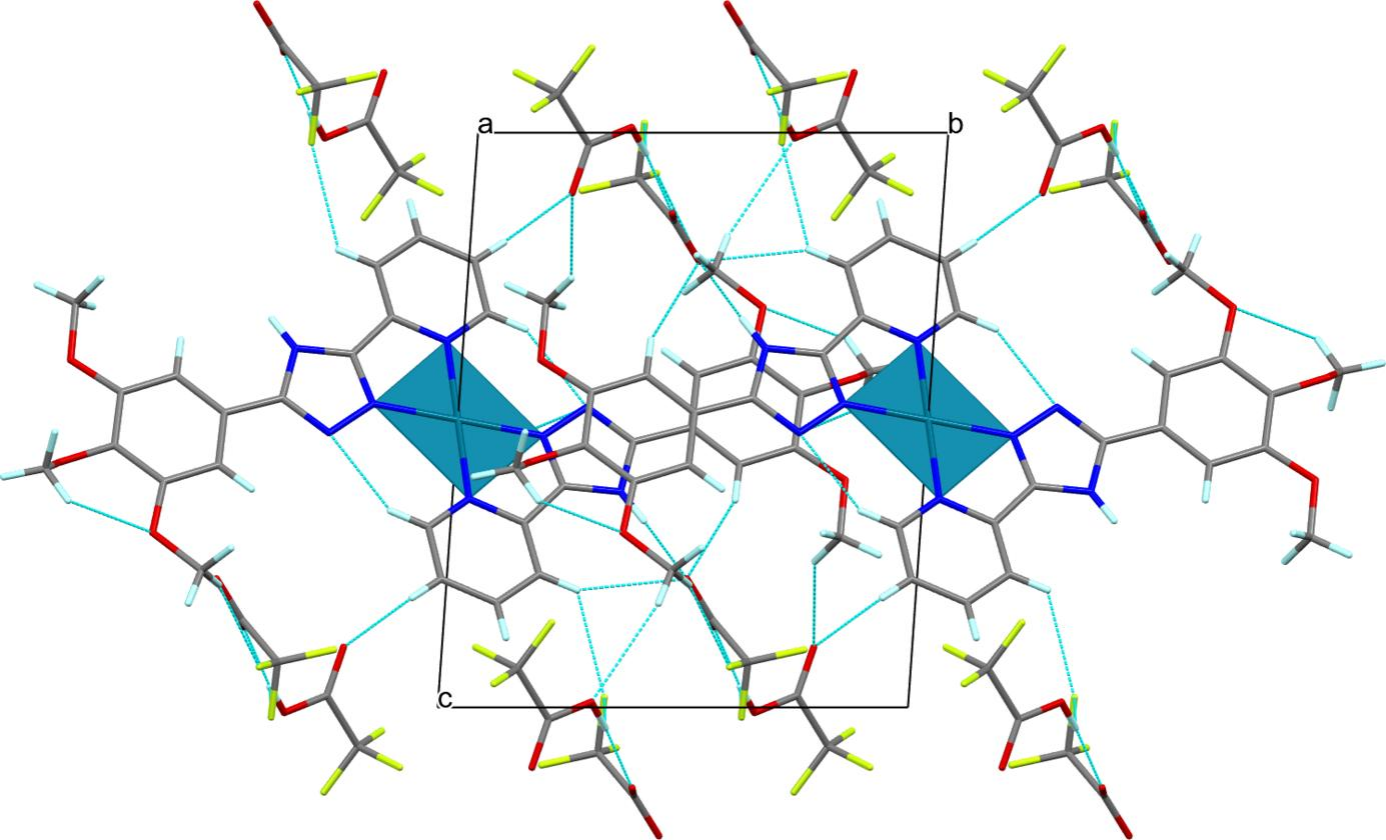
Fragment of the crystal packing of (I) viewed along the *a* axis showing the alternate arrangement of cationic and anionic supra­molecular layers inter­acting through numerous C/N—H⋯F/O contacts. Minor disorder components have been omitted for clarity.

Within the anionic layer, the TFA mol­ecule acts as a proton donor in hydrogen bonding towards the TFA^−^ anion (O6—H6⋯O5; Fig. 2 ▸). The TFA–TFA^−^ pairs stack on both sides of the cationic layers and create a three-dimensional C/N—H⋯F/O hydrogen-bonded network (Fig. 4 ▸). C4—H4*A*⋯O4, N4—H4⋯O4, C9—H9⋯O4 and C14—H14*A*⋯O7 inter­actions between the cation and anion generate inter­connected rings exhibiting 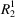 (7) and 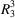 (13) graph-set motifs (Fig. 2 ▸).

## 4. Database survey

More than 1400 crystal structures of metal complexes featuring the 3-(pyridin-2-yl)-1,2,4-triazole backbone having various substituents in the rings are found in the Cambridge Structural Database (CSD, Version 5.45, update of November 2023; Groom *et al.*, 2016 ▸) with the nuclearity up to 24 metal (Co) centres (BIBHUS; Yao *et al.*, 2018 ▸). The only solid-state structure comprising H*L*, the Re^I^ carbonyl [ReBr(H*L*)(CO)_3_]·CH_3_OH (GAMTOG; Kharlova *et al.*, 2017 ▸), differs from (I) in the position of the acidic NH function in the triazole ring. Of nine palladium compounds with 3-(pyridin-2-yl)-1,2,4-triazole derivatives, eight were reported by our research group. In the Pd^II^ complexes, the substituted 3-(pyridin-2-yl)-1,2,4-triazole ligands in the neutral or anionic form coordinate to the metal atom through the pyridine-N and either triazole-N1 (TOFXUK, TOFYAR, TOGNEL, TOGNIP; Zakharchenko *et al.*, 2019 ▸) or triazole-N4 atoms (CAMSUI; Zakhar­chenko *et al.*, 2021*a* ▸). Another example of the N4 protonation is found in hydrogen bis­{2-[3-(pyridin-2-yl)-1,2,4-triazol-1-yl]propano­ate} (CIPCUA; Gallagher *et al.*, 2007 ▸), which is a co-crystal of a neutral mol­ecule and a zwitterion with a proton­ated N4 atom. Most similar, but not isomorphous, to the title compound is [Pd(H*L*′)_2_](CF_3_COO)_2_·4CF_3_COOH with the neutral ligand H*L*′ having a phenyl group instead of the tri­meth­oxy­phenyl substituent in (I), which also crystallizes in the triclinic space group *P* 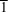 (KEFKUF; Zakharchenko *et al.*, 2021*b* ▸).

## 5. Synthesis and crystallization

The initial complex Pd*L*_2_ was synthesized according to the previously published method (Zakharchenko *et al.*, 2016 ▸). X-ray quality crystals of the title compound were obtained by recrystallization of Pd*L*_2_ from TFA. The compound was characterized by IR and ^1^H NMR spectroscopy; it starts to decompose above 548 K. FT–IR (KBr pellet), ν (cm^−1^): 3434*br*, 3104, 3110, 3010, 2948, 2926, 2850, 1776, 1676, 1638, 1618, 1596, 1490*s*, 1470, 1430, 1292, 1196*s*, 1178*s*, 1130*vs*, 1036, 1006, 842, 796, 726, 704, 598, 568, 524.

**Figure 5:**
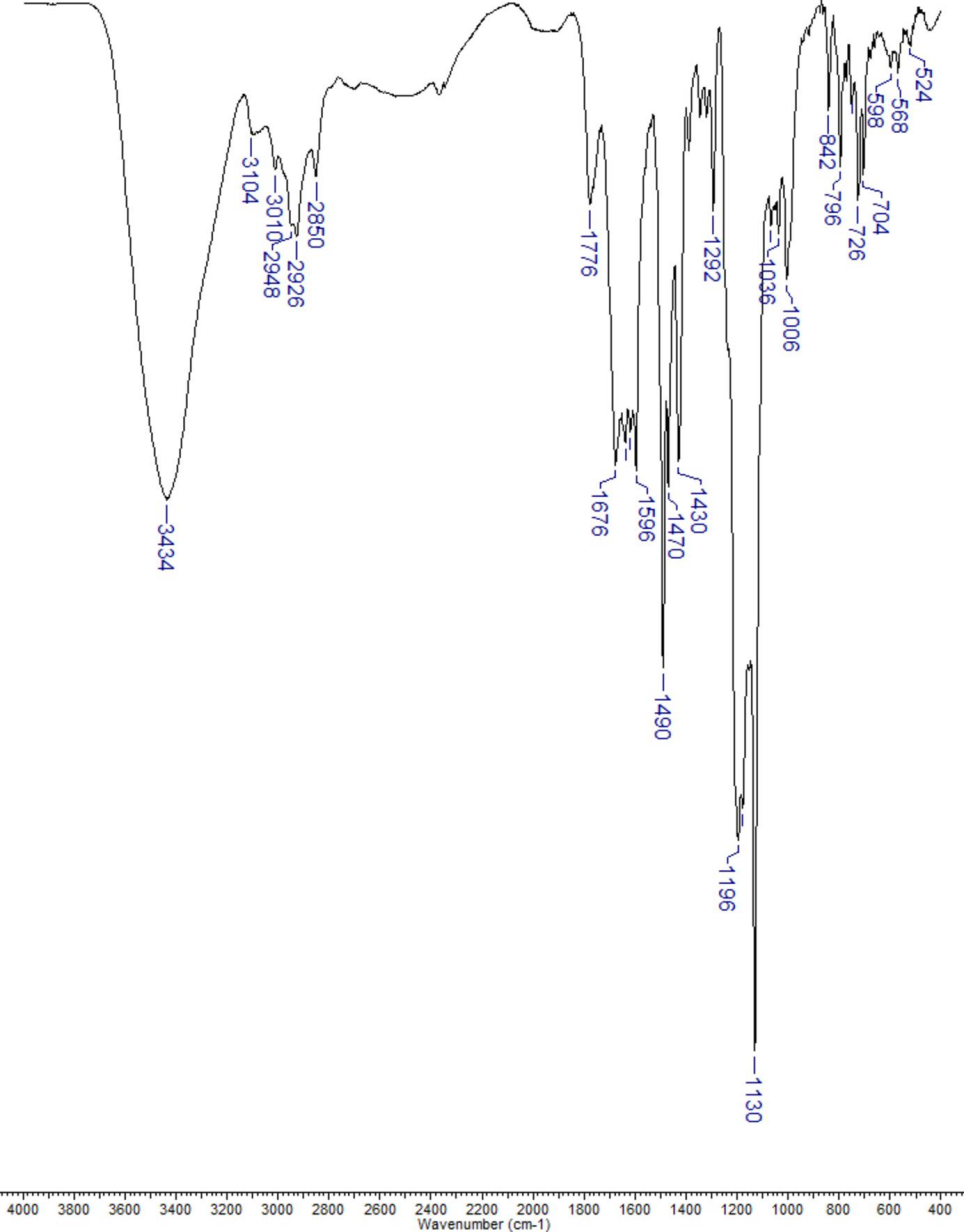
IR spectrum of (I) in the 4000–400 cm^−1^ range.

The IR spectrum of (I) (Fig. 5 ▸) is dominated by peaks associated with tri­fluoro­acetic moieties, which are absent in the spectrum of Pd*L*_2_ (Zakharchenko *et al.*, 2016 ▸). TFA mol­ecules are detected by an intense broad band due to ν(O—H) vibration centred at about 3430 cm^−1^ and a smaller band at 1776 cm^−1^ ascribed to ν(C=O) stretching. Two medium intensity bands observed at 1676 and 1430 cm^−1^ are assigned to ν_as_(COO) and ν_s_(COO) stretching modes of the TFA^−^ anion, respectively. As expected, major absorption peaks at 1196, 1178 and 1130 cm^−1^ are present in the C—F stretching region (1110–1220 cm^−1^). ν(C=N) and ν(C=C) stretching frequencies of the 1,2,4-triazole ligand in the range 1638–1596 cm^−1^ cannot be easily distinguished. Several bands observed above and below 3000 cm^−1^ are assigned to aromatic and methyl group ν(C—H) vibrations, respectively. A low intensity broad absorption at 3104 cm^−1^ can be ascribed to ν(N—H) stretching of the hydrogen-bonded N4H group of the triazole ring.

Due to very poor solubility of the title compound in organic solvents, it was not possible to obtain its satisfactory ^1^H NMR spectrum in CDCl_3_. Only the protons of the meth­oxy groups are distinctly observed as two singlets in a 2:1 ratio at 4.01 and 3.96 ppm while the aromatic protons in the 10–7 ppm range were indistinguishable from the background. The presence of TFA mol­ecules and trace amounts of water in the solvent leads to significant broadening of the N4H signal to the point of disappearing in the spectrum. On the contrary, in the ^1^H NMR spectrum of the free H*L* ligand in CDCl_3_ the acidic N2-bound proton appears as a broadened singlet at 13.31 ppm (Zakharchenko *et al.*, 2016 ▸).

## 6. Refinement

Crystal data, data collection and structure refinement details are summarized in Table 3 ▸. Both tri­fluoro­acetic moieties were found to be disordered over two resolvable positions with a refined occupancy ratio of 0.587 (1):0.413 (17) and 0.530 (6):0.470 (6) for the protonated and deprotonated forms, respectively. The disorder was restrained using SIMU and RIGU commands in *SHELXL* for the ten resulting atoms except for C19 and O4 of the tri­fluoro­acetic anion and twelve resulting atoms except for C17 of the tri­fluoro­acetic acid. The four-atom C—COO fragments were restrained to be nearly planar by a FLAT command. Bond distances in the disordered fragments were restrained by the SAME command to be similar in length. Anisotropic displacement parameters were employed for the non-hydrogen atoms. Anisotropic displacement parameters for pairs of the disordered atoms were constrained to be the same. The H atom bound to O was found in difference-Fourier maps, C/N-bound H atoms were included in calculated positions and refined using a riding model with isotropic displacement parameters based on those of the parent atom [C—H = 0.93 Å, N/O—H = 0.86 Å, *U*_iso_(H) = 1.2*U*_eq_C for CH, NH and OH; C—H = 0.96 Å, *U*_iso_(H) = 1.5*U*_eq_C for CH_3_]. Idealised methyl groups were refined as rotating groups.

## Supplementary Material

Crystal structure: contains datablock(s) I. DOI: 10.1107/S205698902400392X/jw2005sup1.cif

Structure factors: contains datablock(s) I. DOI: 10.1107/S205698902400392X/jw2005Isup2.hkl

CCDC reference: 2352083

Additional supporting information:  crystallographic information; 3D view; checkCIF report

## Figures and Tables

**Table 1:** Selected geometric parameters (Å, °)

Pd1—N2	1.991 (2)	Pd1—N1	2.037 (2)
			
N2—Pd1—N1^i^	100.35 (8)	N2—Pd1—N1	79.65 (8)

Symmetry code: (i) 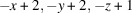 .

**Table 2:** Hydrogen-bond geometry (Å, °)

*D*—H⋯*A*	*D*—H	H⋯*A*	*D*⋯*A*	*D*—H⋯*A*
O6—H6⋯O5	0.84	1.73	2.56 (3)	172
O6*X*—H6*X*⋯F6*X*	0.86	2.27	2.77 (3)	118
N4—H4⋯O4	0.86	1.81	2.655 (3)	166
C15—H15*A*⋯N3^ii^	0.96	2.59	3.334 (5)	134
C15—H15*B*⋯O1	0.96	2.35	2.929 (5)	118
C15—H15*C*⋯O3^iii^	0.96	2.49	3.400 (4)	158
C14—H14*A*⋯O7	0.96	2.67	3.492 (17)	144
C4—H4*A*⋯F6^iv^	0.93	2.58	3.193 (6)	124
C4—H4*A*⋯O4	0.93	2.59	3.425 (4)	150
C9—H9⋯O4	0.93	2.63	3.510 (4)	158
C2—H2⋯O7^v^	0.93	2.44	3.363 (14)	174
C2—H2⋯O7*X* ^v^	0.93	2.57	3.498 (19)	178
C1—H1⋯N3^i^	0.93	2.34	3.146 (3)	145
C16—H16*B*⋯F4^vi^	0.96	2.52	3.400 (7)	152

Symmetry codes: (i) 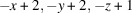 ; (ii) 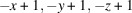 ; (iii) 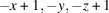 ; (iv) 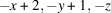 ; (v) 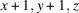 ; (vi) 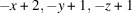 .

**Table 3:** Experimental details

Crystal data	
Chemical formula	[Pd(C_16_H_16_N_4_O_3_)_2_](C_2_F_3_O_2_)_2_·2C_2_HF_3_O_2_
*M*_r_	1185.15
Crystal system, space group	Triclinic, *P* 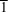
Temperature (K)	293
*a*, *b*, *c* (Å)	8.6173 (4), 10.6265 (6), 13.1312 (4)
α, β, γ (°)	93.384 (4), 98.121 (3), 94.090 (4)
*V* (Å^3^)	1184.46 (9)
*Z*	1
Radiation type	Mo *K*α
μ (mm^−1^)	0.51
Crystal size (mm)	0.4 × 0.3 × 0.3

Data collection	
Diffractometer	Xcalibur, Eos
Absorption correction	Multi-scan (*CrysAlis PRO*; Rigaku OD, 2019 ▸)
*T*_min_, *T*_max_	0.968, 1.000
No. of measured, independent and observed [*I* > 2σ(*I*)] reflections	9462, 5010, 4615
*R*_int_	0.026
(sin θ/λ)_max_ (Å^−1^)	0.633

Refinement	
*R*[*F* ^2^ > 2σ(*F* ^2^)], *wR*(*F* ^2^), *S*	0.042, 0.095, 1.04
No. of reflections	5010
No. of parameters	444
No. of restraints	382
H-atom treatment	H-atom parameters constrained
Δρ_max_, Δρ_min_ (e Å^−3^)	0.39, −0.45

Computer programs: *CrysAlis PRO* (Rigaku OD, 2019 ▸), *SHELXT2014/5* (Sheldrick, 2015*a* ▸), *SHELXL2018/3* (Sheldrick, 2015*b* ▸), *Mercury* (Macrae *et al.*, 2020 ▸) and *OLEX2* (Dolomanov *et al.*, 2009 ▸).

## supplementary crystallographic information

### Crystal data

**Table:**

[Pd(C_16_H_16_N_4_O_3_)_2_](C_2_F_3_O_2_)_2_·2C_2_HF_3_O_2_	*Z* = 1
*M**_r_* = 1185.15	*F*(000) = 596
Triclinic, *P*1	*D*_x_ = 1.662 Mg m^−^^3^
*a* = 8.6173 (4) Å	Mo *K*α radiation, λ = 0.71073 Å
*b* = 10.6265 (6) Å	Cell parameters from 3996 reflections
*c* = 13.1312 (4) Å	θ = 1.6–28.8°
α = 93.384 (4)°	µ = 0.51 mm^−^^1^
β = 98.121 (3)°	*T* = 293 K
γ = 94.090 (4)°	Irregular, clear light yellow
*V* = 1184.46 (9) Å^3^	0.4 × 0.3 × 0.3 mm

### Data collection

**Table:**

Xcalibur, Eos diffractometer	4615 reflections with *I* > 2σ(*I*)
φ and ω scans	*R*_int_ = 0.026
Absorption correction: multi-scan (CrysAlisPro; Rigaku OD, 2019)	θ_max_ = 26.7°, θ_min_ = 1.9°
*T*_min_ = 0.968, *T*_max_ = 1.000	*h* = −10→10
9462 measured reflections	*k* = −13→13
5010 independent reflections	*l* = −16→16

### Refinement

**Table:**

Refinement on *F*^2^	Primary atom site location: dual
Least-squares matrix: full	Hydrogen site location: mixed
*R*[*F*^2^ > 2σ(*F*^2^)] = 0.042	H-atom parameters constrained
*wR*(*F*^2^) = 0.095	*w* = 1/[σ^2^(*F*_o_^2^) + (0.0395*P*)^2^ + 0.5877*P*] where *P* = (*F*_o_^2^ + 2*F*_c_^2^)/3
*S* = 1.04	(Δ/σ)_max_ < 0.001
5010 reflections	Δρ_max_ = 0.39 e Å^−^^3^
444 parameters	Δρ_min_ = −0.45 e Å^−^^3^
382 restraints	

### Special details

**Table:**

Geometry. All esds (except the esd in the dihedral angle between two l.s. planes) are estimated using the full covariance matrix. The cell esds are taken into account individually in the estimation of esds in distances, angles and torsion angles; correlations between esds in cell parameters are only used when they are defined by crystal symmetry. An approximate (isotropic) treatment of cell esds is used for estimating esds involving l.s. planes.

### Fractional atomic coordinates and isotropic or equivalent isotropic displacement parameters (Å^2^)

**Table:**

	*x*	*y*	*z*	*U*_iso_*/*U*_eq_	Occ. (<1)
Pd1	1.000000	1.000000	0.500000	0.02885 (10)	
C19	0.8894 (4)	0.4302 (3)	0.1535 (2)	0.0488 (8)	
C20	0.9655 (9)	0.3470 (9)	0.0791 (6)	0.081 (2)	0.530 (6)
F4	1.1096 (6)	0.3858 (7)	0.0705 (5)	0.109 (2)	0.530 (6)
F5	0.9508 (14)	0.2294 (9)	0.1005 (8)	0.139 (3)	0.530 (6)
F6	0.8973 (9)	0.3527 (8)	−0.0170 (4)	0.108 (2)	0.530 (6)
O5	0.7452 (12)	0.425 (3)	0.140 (2)	0.066 (4)	0.530 (6)
C20X	0.9676 (14)	0.3301 (10)	0.0963 (10)	0.081 (2)	0.470 (6)
F4X	0.9952 (15)	0.3563 (10)	0.0080 (8)	0.134 (3)	0.470 (6)
F5X	1.0921 (8)	0.2898 (7)	0.1463 (6)	0.110 (3)	0.470 (6)
F6X	0.8690 (10)	0.2246 (8)	0.0722 (7)	0.107 (3)	0.470 (6)
O5X	0.7498 (15)	0.441 (3)	0.123 (3)	0.074 (6)	0.470 (6)
O4	0.9779 (3)	0.4896 (2)	0.22379 (16)	0.0580 (6)	
C17	0.4530 (4)	0.2377 (4)	0.0206 (3)	0.0643 (10)	
C18	0.3492 (9)	0.1637 (9)	−0.0678 (6)	0.095 (2)	0.587 (17)
F1	0.3053 (15)	0.2296 (12)	−0.1475 (7)	0.136 (3)	0.587 (17)
F2	0.4262 (13)	0.0721 (12)	−0.1043 (11)	0.143 (3)	0.587 (17)
F3	0.2162 (9)	0.1158 (12)	−0.0410 (7)	0.118 (3)	0.587 (17)
O7	0.442 (2)	0.2093 (17)	0.1046 (6)	0.100 (5)	0.587 (17)
O6	0.5404 (17)	0.3257 (15)	−0.0093 (13)	0.094 (5)	0.587 (17)
H6	0.613798	0.359574	0.035314	0.113*	0.587 (17)
C18X	0.3438 (13)	0.1690 (10)	−0.0676 (7)	0.101 (3)	0.413 (17)
F1X	0.4166 (17)	0.1293 (19)	−0.1438 (10)	0.125 (4)	0.413 (17)
F2X	0.2443 (16)	0.2475 (14)	−0.1086 (13)	0.132 (4)	0.413 (17)
F3X	0.278 (2)	0.0643 (15)	−0.0378 (12)	0.141 (4)	0.413 (17)
O7X	0.428 (3)	0.241 (2)	0.1062 (8)	0.078 (4)	0.413 (17)
O6X	0.574 (2)	0.288 (2)	−0.0120 (18)	0.081 (5)	0.413 (17)
H6X	0.646340	0.324285	0.033784	0.097*	0.413 (17)
O1	0.5903 (3)	0.3676 (2)	0.69590 (17)	0.0607 (7)	
O2	0.6094 (3)	0.15652 (19)	0.57418 (17)	0.0506 (5)	
O3	0.7086 (3)	0.16746 (19)	0.39566 (16)	0.0543 (6)	
N4	0.9103 (3)	0.6437 (2)	0.37512 (15)	0.0306 (5)	
H4	0.916011	0.589624	0.324689	0.037*	
N3	0.8561 (3)	0.7280 (2)	0.52237 (16)	0.0329 (5)	
N2	0.9308 (3)	0.81692 (19)	0.47212 (15)	0.0300 (5)	
N1	1.0785 (3)	0.9649 (2)	0.36266 (15)	0.0311 (5)	
C10	0.7119 (3)	0.2797 (3)	0.4517 (2)	0.0386 (6)	
C15	0.4529 (4)	0.1298 (4)	0.5844 (4)	0.0795 (13)	
H15A	0.387704	0.144900	0.521519	0.119*	
H15B	0.426681	0.183175	0.640017	0.119*	
H15C	0.435939	0.042788	0.598890	0.119*	
C7	0.8445 (3)	0.6231 (2)	0.46214 (19)	0.0303 (6)	
C12	0.6508 (3)	0.3838 (3)	0.6071 (2)	0.0409 (7)	
C14	0.7558 (5)	0.1699 (3)	0.2963 (2)	0.0638 (10)	
H14A	0.687992	0.219861	0.253864	0.096*	
H14B	0.749120	0.085221	0.265300	0.096*	
H14C	0.862275	0.206258	0.302706	0.096*	
C4	1.0995 (3)	0.8044 (3)	0.2315 (2)	0.0388 (7)	
H4A	1.077270	0.721198	0.204347	0.047*	
C9	0.7732 (3)	0.3943 (3)	0.4228 (2)	0.0365 (6)	
H9	0.813638	0.398525	0.360914	0.044*	
C2	1.2150 (4)	1.0120 (3)	0.2234 (2)	0.0497 (8)	
H2	1.272046	1.071011	0.190832	0.060*	
C13	0.7108 (3)	0.4976 (3)	0.5783 (2)	0.0382 (6)	
H13	0.709298	0.571194	0.620054	0.046*	
C1	1.1601 (4)	1.0467 (3)	0.3140 (2)	0.0403 (7)	
H1	1.180841	1.129773	0.341708	0.048*	
C3	1.1847 (4)	0.8902 (3)	0.1821 (2)	0.0492 (8)	
H3	1.221162	0.865472	0.121268	0.059*	
C5	1.0485 (3)	0.8437 (2)	0.32096 (18)	0.0293 (5)	
C11	0.6520 (3)	0.2734 (3)	0.5440 (2)	0.0384 (6)	
C8	0.7734 (3)	0.5023 (2)	0.4872 (2)	0.0322 (6)	
C16	0.5794 (5)	0.4776 (4)	0.7613 (3)	0.0674 (11)	
H16A	0.519452	0.536517	0.723050	0.101*	
H16B	0.683090	0.516035	0.786165	0.101*	
H16C	0.528455	0.454170	0.818594	0.101*	
C6	0.9639 (3)	0.7659 (2)	0.38500 (18)	0.0292 (5)	

### Atomic displacement parameters (Å^2^)

**Table:**

	*U*^11^	*U*^22^	*U*^33^	*U*^12^	*U*^13^	*U*^23^
Pd1	0.04096 (18)	0.01711 (15)	0.02989 (16)	−0.00198 (11)	0.01369 (12)	−0.00184 (10)
C19	0.064 (2)	0.0396 (18)	0.0431 (17)	−0.0077 (16)	0.0198 (16)	−0.0102 (14)
C20	0.110 (4)	0.077 (4)	0.058 (4)	0.005 (4)	0.035 (4)	−0.034 (4)
F4	0.074 (3)	0.144 (5)	0.105 (4)	0.008 (3)	0.032 (3)	−0.064 (4)
F5	0.189 (8)	0.086 (5)	0.153 (7)	0.043 (5)	0.057 (6)	−0.014 (5)
F6	0.111 (5)	0.153 (5)	0.052 (3)	−0.007 (4)	0.021 (3)	−0.057 (3)
O5	0.059 (5)	0.079 (9)	0.057 (6)	−0.014 (4)	0.013 (4)	−0.010 (5)
C20X	0.108 (5)	0.063 (5)	0.070 (5)	0.000 (4)	0.023 (4)	−0.028 (4)
F4X	0.187 (8)	0.130 (5)	0.101 (6)	0.022 (6)	0.079 (6)	−0.014 (5)
F5X	0.103 (4)	0.089 (5)	0.133 (5)	0.049 (4)	0.001 (4)	−0.046 (4)
F6X	0.124 (6)	0.067 (4)	0.115 (5)	−0.011 (4)	0.001 (4)	−0.058 (3)
O5X	0.076 (7)	0.072 (9)	0.073 (12)	−0.012 (5)	0.021 (5)	−0.015 (8)
O4	0.0708 (16)	0.0521 (15)	0.0483 (13)	−0.0024 (12)	0.0127 (11)	−0.0217 (11)
C17	0.053 (2)	0.075 (3)	0.063 (2)	−0.0078 (19)	0.0090 (18)	0.004 (2)
C18	0.079 (5)	0.117 (5)	0.083 (5)	−0.024 (4)	0.013 (4)	−0.019 (4)
F1	0.124 (6)	0.186 (7)	0.083 (4)	−0.025 (5)	−0.024 (4)	0.010 (5)
F2	0.151 (6)	0.131 (7)	0.132 (7)	−0.006 (5)	0.001 (5)	−0.058 (5)
F3	0.068 (4)	0.142 (7)	0.131 (4)	−0.045 (4)	0.004 (3)	−0.010 (5)
O7	0.099 (7)	0.123 (11)	0.075 (6)	−0.035 (7)	0.006 (5)	0.040 (6)
O6	0.097 (8)	0.116 (9)	0.059 (5)	−0.046 (7)	−0.002 (5)	0.022 (6)
C18X	0.086 (6)	0.122 (6)	0.088 (6)	−0.023 (6)	0.010 (5)	−0.019 (5)
F1X	0.125 (6)	0.151 (9)	0.089 (6)	−0.018 (7)	0.017 (5)	−0.042 (6)
F2X	0.087 (6)	0.176 (7)	0.115 (7)	0.012 (6)	−0.031 (5)	−0.022 (6)
F3X	0.132 (8)	0.129 (8)	0.147 (7)	−0.064 (6)	0.017 (7)	−0.008 (6)
O7X	0.099 (8)	0.076 (8)	0.065 (7)	−0.007 (6)	0.039 (6)	−0.012 (5)
O6X	0.062 (6)	0.116 (12)	0.057 (6)	−0.028 (7)	0.013 (5)	−0.013 (7)
O1	0.0919 (18)	0.0384 (13)	0.0582 (14)	−0.0097 (12)	0.0406 (13)	0.0027 (10)
O2	0.0538 (13)	0.0281 (11)	0.0716 (15)	−0.0050 (10)	0.0154 (11)	0.0121 (10)
O3	0.0882 (17)	0.0208 (11)	0.0522 (13)	−0.0098 (11)	0.0160 (11)	−0.0074 (9)
N4	0.0412 (13)	0.0206 (11)	0.0288 (11)	−0.0052 (9)	0.0084 (9)	−0.0058 (8)
N3	0.0463 (13)	0.0196 (11)	0.0345 (11)	−0.0037 (9)	0.0148 (10)	0.0010 (9)
N2	0.0410 (13)	0.0181 (11)	0.0323 (11)	−0.0024 (9)	0.0139 (9)	−0.0017 (8)
N1	0.0429 (13)	0.0206 (11)	0.0311 (11)	−0.0022 (9)	0.0131 (9)	−0.0012 (8)
C10	0.0488 (17)	0.0232 (14)	0.0419 (15)	−0.0044 (12)	0.0057 (13)	−0.0013 (11)
C15	0.054 (2)	0.042 (2)	0.143 (4)	−0.0146 (17)	0.026 (2)	0.006 (2)
C7	0.0371 (15)	0.0202 (13)	0.0332 (13)	−0.0012 (11)	0.0068 (11)	−0.0010 (10)
C12	0.0465 (17)	0.0322 (16)	0.0460 (16)	−0.0022 (13)	0.0162 (13)	0.0044 (13)
C14	0.105 (3)	0.0382 (19)	0.0460 (19)	−0.0013 (19)	0.0120 (18)	−0.0093 (15)
C4	0.0528 (18)	0.0307 (15)	0.0322 (14)	−0.0040 (13)	0.0113 (12)	−0.0064 (11)
C9	0.0494 (17)	0.0244 (14)	0.0351 (14)	−0.0046 (12)	0.0087 (12)	−0.0005 (11)
C2	0.071 (2)	0.0403 (18)	0.0423 (16)	−0.0079 (16)	0.0301 (15)	0.0003 (13)
C13	0.0506 (17)	0.0238 (14)	0.0407 (15)	−0.0027 (12)	0.0135 (13)	−0.0026 (11)
C1	0.0583 (19)	0.0241 (14)	0.0403 (15)	−0.0054 (13)	0.0197 (13)	−0.0011 (11)
C3	0.070 (2)	0.0453 (19)	0.0349 (15)	−0.0060 (16)	0.0272 (15)	−0.0070 (13)
C5	0.0341 (14)	0.0248 (13)	0.0289 (12)	0.0010 (11)	0.0055 (10)	0.0003 (10)
C11	0.0420 (16)	0.0239 (14)	0.0488 (16)	−0.0052 (12)	0.0067 (13)	0.0067 (12)
C8	0.0391 (15)	0.0216 (13)	0.0352 (14)	−0.0029 (11)	0.0061 (11)	0.0011 (10)
C16	0.093 (3)	0.057 (2)	0.059 (2)	0.001 (2)	0.040 (2)	−0.0033 (18)
C6	0.0369 (14)	0.0209 (13)	0.0291 (12)	−0.0016 (10)	0.0071 (10)	−0.0033 (10)

### Geometric parameters (Å, º)

**Table:**

Pd1—N2	1.991 (2)	N4—H4	0.8600
Pd1—N2^i^	1.991 (2)	N4—C7	1.367 (3)
Pd1—N1	2.037 (2)	N4—C6	1.340 (3)
Pd1—N1^i^	2.037 (2)	N3—N2	1.360 (3)
C19—C20	1.526 (9)	N3—C7	1.317 (3)
C19—O5	1.227 (11)	N2—C6	1.313 (3)
C19—C20X	1.517 (11)	N1—C1	1.328 (3)
C19—O5X	1.231 (13)	N1—C5	1.362 (3)
C19—O4	1.223 (4)	C10—C9	1.388 (4)
C20—F4	1.303 (7)	C10—C11	1.385 (4)
C20—F5	1.298 (7)	C15—H15A	0.9600
C20—F6	1.321 (7)	C15—H15B	0.9600
C20X—F4X	1.258 (12)	C15—H15C	0.9600
C20X—F5X	1.290 (12)	C7—C8	1.458 (3)
C20X—F6X	1.351 (11)	C12—C13	1.376 (4)
C17—C18	1.504 (7)	C12—C11	1.397 (4)
C17—O7	1.177 (7)	C14—H14A	0.9600
C17—O6	1.273 (7)	C14—H14B	0.9600
C17—C18X	1.501 (8)	C14—H14C	0.9600
C17—O7X	1.174 (8)	C4—H4A	0.9300
C17—O6X	1.275 (8)	C4—C3	1.378 (4)
C18—F1	1.320 (6)	C4—C5	1.365 (3)
C18—F2	1.321 (6)	C9—H9	0.9300
C18—F3	1.323 (6)	C9—C8	1.384 (4)
O6—H6	0.8428	C2—H2	0.9300
C18X—F1X	1.319 (7)	C2—C1	1.380 (4)
C18X—F2X	1.321 (7)	C2—C3	1.366 (4)
C18X—F3X	1.319 (7)	C13—H13	0.9300
O6X—H6X	0.8555	C13—C8	1.383 (4)
O1—C12	1.357 (3)	C1—H1	0.9300
O1—C16	1.425 (4)	C3—H3	0.9300
O2—C15	1.385 (4)	C5—C6	1.446 (3)
O2—C11	1.367 (3)	C16—H16A	0.9600
O3—C10	1.360 (3)	C16—H16B	0.9600
O3—C14	1.421 (4)	C16—H16C	0.9600
			
N2—Pd1—N2^i^	180.00 (12)	O3—C10—C9	124.2 (3)
N2—Pd1—N1^i^	100.35 (8)	O3—C10—C11	115.4 (2)
N2^i^—Pd1—N1	100.35 (8)	C11—C10—C9	120.4 (3)
N2^i^—Pd1—N1^i^	79.65 (8)	O2—C15—H15A	109.5
N2—Pd1—N1	79.65 (8)	O2—C15—H15B	109.5
N1^i^—Pd1—N1	180.00 (12)	O2—C15—H15C	109.5
O5—C19—C20	116.4 (14)	H15A—C15—H15B	109.5
O5X—C19—C20X	116.7 (16)	H15A—C15—H15C	109.5
O4—C19—C20	116.6 (4)	H15B—C15—H15C	109.5
O4—C19—O5	126.9 (14)	N4—C7—C8	125.5 (2)
O4—C19—C20X	113.6 (6)	N3—C7—N4	110.5 (2)
O4—C19—O5X	129.6 (17)	N3—C7—C8	123.9 (2)
F4—C20—C19	114.3 (6)	O1—C12—C13	124.8 (3)
F4—C20—F6	100.3 (7)	O1—C12—C11	115.1 (3)
F5—C20—C19	110.6 (7)	C13—C12—C11	120.0 (2)
F5—C20—F4	112.8 (8)	O3—C14—H14A	109.5
F5—C20—F6	107.0 (9)	O3—C14—H14B	109.5
F6—C20—C19	111.1 (6)	O3—C14—H14C	109.5
F4X—C20X—C19	115.2 (10)	H14A—C14—H14B	109.5
F4X—C20X—F5X	108.9 (11)	H14A—C14—H14C	109.5
F4X—C20X—F6X	100.6 (11)	H14B—C14—H14C	109.5
F5X—C20X—C19	116.1 (9)	C3—C4—H4A	120.6
F5X—C20X—F6X	103.9 (9)	C5—C4—H4A	120.6
F6X—C20X—C19	110.5 (9)	C5—C4—C3	118.8 (3)
O7—C17—C18	117.9 (10)	C10—C9—H9	120.4
O7—C17—O6	129.6 (11)	C8—C9—C10	119.1 (2)
O6—C17—C18	112.5 (9)	C8—C9—H9	120.4
O7X—C17—C18X	123.2 (13)	C1—C2—H2	120.3
O7X—C17—O6X	126.8 (15)	C3—C2—H2	120.3
O6X—C17—C18X	110.0 (12)	C3—C2—C1	119.4 (3)
F1—C18—C17	114.7 (7)	C12—C13—H13	120.1
F1—C18—F2	105.2 (8)	C12—C13—C8	119.8 (3)
F1—C18—F3	104.8 (8)	C8—C13—H13	120.1
F2—C18—C17	109.5 (7)	N1—C1—C2	121.9 (3)
F2—C18—F3	109.9 (8)	N1—C1—H1	119.0
F3—C18—C17	112.4 (6)	C2—C1—H1	119.0
C17—O6—H6	116.1	C4—C3—H3	120.3
F1X—C18X—C17	113.1 (9)	C2—C3—C4	119.4 (3)
F1X—C18X—F2X	105.1 (10)	C2—C3—H3	120.3
F1X—C18X—F3X	103.9 (10)	N1—C5—C4	122.1 (2)
F2X—C18X—C17	109.2 (8)	N1—C5—C6	111.5 (2)
F3X—C18X—C17	110.4 (9)	C4—C5—C6	126.3 (2)
F3X—C18X—F2X	114.9 (12)	O2—C11—C10	117.9 (3)
C17—O6X—H6X	116.5	O2—C11—C12	122.1 (3)
C12—O1—C16	117.7 (2)	C10—C11—C12	119.6 (2)
C11—O2—C15	117.5 (3)	C9—C8—C7	120.7 (2)
C10—O3—C14	117.7 (2)	C13—C8—C7	118.3 (2)
C7—N4—H4	127.3	C13—C8—C9	120.9 (2)
C6—N4—H4	127.3	O1—C16—H16A	109.5
C6—N4—C7	105.5 (2)	O1—C16—H16B	109.5
C7—N3—N2	105.36 (19)	O1—C16—H16C	109.5
N3—N2—Pd1	135.44 (16)	H16A—C16—H16B	109.5
C6—N2—Pd1	114.75 (16)	H16A—C16—H16C	109.5
C6—N2—N3	109.8 (2)	H16B—C16—H16C	109.5
C1—N1—Pd1	126.23 (18)	N4—C6—C5	132.4 (2)
C1—N1—C5	118.4 (2)	N2—C6—N4	108.8 (2)
C5—N1—Pd1	115.29 (16)	N2—C6—C5	118.8 (2)
			
Pd1—N2—C6—N4	−179.45 (16)	N3—C7—C8—C9	−176.4 (3)
Pd1—N2—C6—C5	1.7 (3)	N3—C7—C8—C13	1.6 (4)
Pd1—N1—C1—C2	176.7 (2)	N2—N3—C7—N4	0.1 (3)
Pd1—N1—C5—C4	−177.2 (2)	N2—N3—C7—C8	179.1 (2)
Pd1—N1—C5—C6	0.6 (3)	N1—C5—C6—N4	179.9 (3)
O5—C19—C20—F4	156.2 (17)	N1—C5—C6—N2	−1.5 (3)
O5—C19—C20—F5	−75.1 (18)	C10—C9—C8—C7	176.6 (3)
O5—C19—C20—F6	43.6 (18)	C10—C9—C8—C13	−1.3 (4)
O5X—C19—C20X—F4X	73 (2)	C15—O2—C11—C10	−118.1 (4)
O5X—C19—C20X—F5X	−158 (2)	C15—O2—C11—C12	68.1 (4)
O5X—C19—C20X—F6X	−40 (2)	C7—N4—C6—N2	−0.8 (3)
O4—C19—C20—F4	−27.5 (8)	C7—N4—C6—C5	177.9 (3)
O4—C19—C20—F5	101.2 (8)	C7—N3—N2—Pd1	179.9 (2)
O4—C19—C20—F6	−140.1 (6)	C7—N3—N2—C6	−0.6 (3)
O4—C19—C20X—F4X	−106.0 (11)	C12—C13—C8—C7	−176.4 (3)
O4—C19—C20X—F5X	22.9 (12)	C12—C13—C8—C9	1.6 (4)
O4—C19—C20X—F6X	140.8 (9)	C14—O3—C10—C9	−6.8 (5)
O7—C17—C18—F1	147.3 (16)	C14—O3—C10—C11	174.6 (3)
O7—C17—C18—F2	−94.6 (16)	C4—C5—C6—N4	−2.4 (5)
O7—C17—C18—F3	27.8 (18)	C4—C5—C6—N2	176.2 (3)
O6—C17—C18—F1	−31.6 (16)	C9—C10—C11—O2	−172.8 (3)
O6—C17—C18—F2	86.4 (15)	C9—C10—C11—C12	1.2 (5)
O6—C17—C18—F3	−151.2 (15)	C13—C12—C11—O2	172.9 (3)
O7X—C17—C18X—F1X	−151.8 (19)	C13—C12—C11—C10	−0.8 (5)
O7X—C17—C18X—F2X	91.5 (19)	C1—N1—C5—C4	0.1 (4)
O7X—C17—C18X—F3X	−36 (2)	C1—N1—C5—C6	177.9 (2)
O6X—C17—C18X—F1X	27 (2)	C1—C2—C3—C4	0.2 (5)
O6X—C17—C18X—F2X	−89.8 (19)	C3—C4—C5—N1	0.2 (4)
O6X—C17—C18X—F3X	142.9 (19)	C3—C4—C5—C6	−177.2 (3)
O1—C12—C13—C8	178.3 (3)	C3—C2—C1—N1	0.1 (5)
O1—C12—C11—O2	−6.0 (4)	C5—N1—C1—C2	−0.3 (4)
O1—C12—C11—C10	−179.7 (3)	C5—C4—C3—C2	−0.3 (5)
O3—C10—C9—C8	−178.6 (3)	C11—C10—C9—C8	−0.1 (4)
O3—C10—C11—O2	5.8 (4)	C11—C12—C13—C8	−0.5 (5)
O3—C10—C11—C12	179.8 (3)	C16—O1—C12—C13	3.9 (5)
N4—C7—C8—C9	2.5 (4)	C16—O1—C12—C11	−177.2 (3)
N4—C7—C8—C13	−179.5 (3)	C6—N4—C7—N3	0.4 (3)
N3—N2—C6—N4	0.9 (3)	C6—N4—C7—C8	−178.5 (2)
N3—N2—C6—C5	−178.0 (2)		

Symmetry code: (i) −*x*+2, −*y*+2, −*z*+1.

### Hydrogen-bond geometry (Å, º)

**Table:**

*D*—H···*A*	*D*—H	H···*A*	*D*···*A*	*D*—H···*A*
O6—H6···F6	0.84	2.63	3.085 (15)	115
O6—H6···O5	0.84	1.73	2.56 (3)	172
O6*X*—H6*X*···F6*X*	0.86	2.27	2.77 (3)	118
O6*X*—H6*X*···O5*X*	0.86	1.76	2.58 (4)	160
N4—H4···O4	0.86	1.81	2.655 (3)	166
C15—H15*A*···N3^ii^	0.96	2.59	3.334 (5)	134
C15—H15*B*···O1	0.96	2.35	2.929 (5)	118
C15—H15*C*···O3^iii^	0.96	2.49	3.400 (4)	158
C14—H14*A*···O7	0.96	2.67	3.492 (17)	144
C4—H4*A*···F6^iv^	0.93	2.58	3.193 (6)	124
C4—H4*A*···O4	0.93	2.59	3.425 (4)	150
C9—H9···O4	0.93	2.63	3.510 (4)	158
C2—H2···O7^v^	0.93	2.44	3.363 (14)	174
C2—H2···O7*X*^v^	0.93	2.57	3.498 (19)	178
C1—H1···N3^i^	0.93	2.34	3.146 (3)	145
C16—H16*B*···F4^vi^	0.96	2.52	3.400 (7)	152

Symmetry codes: (i) −*x*+2, −*y*+2, −*z*+1; (ii) −*x*+1, −*y*+1, −*z*+1; (iii) −*x*+1, −*y*, −*z*+1; (iv) −*x*+2, −*y*+1, −*z*; (v) *x*+1, *y*+1, *z*; (vi) −*x*+2, −*y*+1, −*z*+1.
